# Event-based record linkage in health and aged care services data: a methodological innovation

**DOI:** 10.1186/1472-6963-7-154

**Published:** 2007-09-25

**Authors:** Rosemary Karmel, Diane Gibson

**Affiliations:** 1 Australian Institute of Health and Welfare, 26 Thynne Street, Fern Hill Park, Canberra, Australia

## Abstract

**Background:**

The interface between acute hospital care and residential aged care has long been recognised as an important issue in aged care services research in Australia. However, existing national data provide very poor information on the movements of clients between the two sectors. Nevertheless, there are national data sets which separately contain data on individuals' hospital episodes and stays in residential aged care, so that linking the two data sets–if feasible–would provide a valuable resource for examining relationships between the two sectors. As neither name nor common person identifiers are available on the data sets, other information needs to be used to link events relating to inter-sector movement.

**Methods:**

Event-based matching using limited demographic data in conjunction with event dates to match events in two data sets provides a possible method for linking related events. The authors develop a statistical model for examining the likely prevalence of false matches, and consequently the number of true matches, among achieved matches when using anonymous event-based record linkage to identify transition events.

**Results:**

Theoretical analysis shows that for event-based matching the prevalence of false matches among achieved matches (a) declines as the events of interest become rarer, (b) declines as the number of matches increases, and (c) increases with the size of the population within which matching is taking place. The method also facilitates the examination of the trade-off between false matches and missed matches when relaxing or tightening linkage criteria.

**Conclusion:**

Event-based record linkage is a method for linking related transition events using event dates and basic demographic variables (other than name or person identifier). The likely extent of false links among achieved links depends on the two event rates, the match rate and population size. Knowing these, it is possible to gauge whether, for a particular study, event-based linkage could provide a useful tool for examining movements. Analysis shows that there is a range of circumstances in which event-based record linkage could be applied to two event-level databases to generate a linked database useful for transition analysis.

## Background

In Australia, hospital use increases with age, both in terms of numbers of visits and days in hospital, and it is roughly estimated that around 3% of hospital separations for older people (65+) involve transfers to residential aged care [1-3]. On the other hand, perhaps as many as 50% of residential aged care admissions come from hospital. In addition, many aged care residents have periods in hospital and about 5% of residential aged care periods end with the resident being discharged to hospital [4].

The interface between acute hospital care and residential aged care has long been recognised as an important issue in aged care services research in Australia [5,6]. The policy significance of cross-sector movement was brought to the fore in 2001 with the establishment by the Australian Health Ministers' Advisory Council of the Care of Older Australians Working Group (COAWG) (reformed as the Health Care of Older Australians Standing Committee in 2005) 'in recognition of the need to improve the interface between acute hospital care, community care and residential aged care to ensure that older people receive the most appropriate care' [7]. Between 2002 and 2004 COAWG sponsored a research program which led to the release of a number of reports looking into different aspects of the interface [8-14].

Despite general recognition of the importance of the relationship between the sectors, existing national data provide very poor information on the movements of clients between the residential and acute care sectors. However, there are national data sets based on information collected primarily for administrative purposes which separately contain data on hospital episodes and residential aged care use, so that linking the two data sets–if feasible–would provide a valuable resource for examining relationships between the two sectors.

Record linkage is a powerful tool for combining information and extending the utility of data sets beyond their individual boundaries. It is a technique that has been successfully employed in a range of fields to exploit currently available data to address a range of issues [15-20]. In addition, there is an emerging recognition that data linkage between existing data sets greatly facilitates investigations into many issues for which it is very difficult and/or expensive to obtain purpose-specific data [21,22].

Two methods are typically used when trying to match people's information on one data set with their data on another: name-based matching (often probabilistic) or matching using a unique person identifier [23-26], with data handling protocols being used to protect confidentiality and privacy [27,28]. In the current case, the absence of both name and a common person identifier on both of the data sets precludes linking data using either of these approaches. However, a range of other data items is common to both data sets, and so the challenge is to determine whether there is sufficient information on the two data sets to allow reliable record linkage to generate a linked database on individuals who move from hospital to the residential care sector which could then be used for analysis of patterns of movements. Such anonymous record linkage, without access to name information–anonymised or otherwise–or a unique person identifier, has been used successfully before in a number of scenarios [29-32]. These studies commonly include matching by date of birth and sex within region, in conjunction with study-specific non-name variables.

In 2001, the Australian Institute of Health and Welfare (AIHW) commenced work on a project aimed at exploring statistically the interface between residential aged care services and the acute hospital sector by linking currently available administrative by-product data. The Institute began by carrying out a feasibility study to trial a data linkage methodology based on event dates and available demographic variables [13]. Results from the feasibility study suggest that this statistical linkage methodology successfully generates a set of linked client records which could be used to examine the relationship between the hospital and residential aged care sectors.

The strategy investigated in the feasibility study linked related events by matching admissions into residential aged care and any hospital stays by permanent aged care residents with a hospital separation using data on birth date, sex, region of usual residence, and hospital separation/residential aged care admission or re-entry event dates. As with other record linkage strategies, the matching process aims to link records for the same person, rather than simply linking people who are similar in a range of demographic variables–as is done in statistical matching [33].

When linking records four outcomes are possible: a true link, no link (true negative), a false link (false positive) and a missed link (false negative). In the event-based strategy, false links can be caused by several individuals (either leaving hospital or entering residential aged care) having the same demographic data so that unrelated events are erroneously linked. In addition, inconsistencies in the matching data caused by either transcription or reporting discrepancies may cause false links to be made or lead to missing some links [34-36].

Using statistical theory, this paper focuses on estimating the extent of false links, and therefore also of true links, among achieved matches due to the non-uniqueness of demographic data. Findings from this analysis are then used to establish under what circumstances an event-based strategy could be useful. When there are inconsistencies in the data used for matching, relaxing the match criteria can result in the identification of previously-missed links. Without comparing the results of a linkage strategy with those from a gold standard it is not possible to gauge the full extent of missed matches. However, by comparing the theoretically estimated numbers of false and true links among matches achieved using linkage criteria of varying degrees of strictness the trade-off between the two can be explored. Such information is very valuable in the common situation where there is no relevant gold standard. Thus, while the theoretical approach by itself cannot provide an estimate of the total number of missed matches for a particular strategy, it provides a practical tool for estimating the gains and losses in moving from one linkage strategy to another, and therefore can assist in developing a preferred linkage strategy. A simple example using the theoretically estimated false match rate to identify a preferred match strategy is given in the Discussion.

As emphasised by Roos and Wajda, 'knowing whether or not a linkage is likely to be feasible is important' [37]. If the number of false matches due to similar individuals having similar events is likely to make up a high proportion of achieved matches where accurate recording of data is assumed, then there is limited value in progressing further with the event-based method to resource-intensive studies which examine empirically the extent of all types of errors. If the proposed method is found to produce an acceptable level of data linkage on the basis of tests derived from statistical theory, then subsequent studies can be used to further determine if and for what purposes the proposed linkage strategy could be used (for example, examining patterns of service use for policy-related research versus clinical research).

It is important to note that linkage studies involving the use of administrative data should only be undertaken with appropriate recognition of ethical and privacy considerations, and with the approval of an ethics committee. If the linkage method outlined in this article is found to be practical for a particular study, then–as for any such linkage studies using data initially gathered for other purposes and so without participant consent for the proposed linkage–agreement and clearance would need to be obtained from a properly constituted ethics committee or ethics review board. In our own work, the authors of this paper also adhere to clearly stated data-handling linkage protocols that protect the privacy of individuals (see, for example, [27] and [28]).

## Methods

In the context of the acute care-aged care interface, the purpose of the event-based linkage strategy is to match hospital separations to entries into residential aged care for people who are (a) admitted into residential aged care following a hospital episode, or (b) already permanent aged care residents. Intuitively, among people who usually live in a particular small region we would expect there to be few cases in which a person leaving hospital on a particular day has the same date of birth as someone else of the same sex from that region entering residential aged care on that day. That is, we would expect only a small number of false matches due to identical demographic characteristics. Whether or not such chance matches make up a sizeable proportion of all matches, and therefore affect the utility of the linked data, can be seen by comparing the number of chance (and therefore false) matches with the total number of achieved matches; that is, by estimating a false match rate per hospital separation date (termed the 'false match rate').

Assuming accurate recording of linkage data, the number of false matches among achieved matches can be estimated by considering the probability of a match between a hospital separation and a residential aged care entry purely by chance due to the distribution of birth dates. To make the estimation of the number of false matches tractable for the current application, two simplifying assumptions are made:

• that birth dates are spread across a selected time interval (of length β days), and

• that birth dates have a uniform distribution across the selected time interval.

The daily expected number of chance matches C due to coincident birth dates when comparing *m *hospital separations (for a particular day) with *k *candidate residential aged care admissions or returns is then estimated by:

C = Estimated number of chance matches

= (Probability of a single hospital separation having the same birth date as a candidate aged care admission or return) × (number of comparisons)

= 1/(Total number of possible birth dates)

× (Number of possibly matching residential aged care entries)

× (Number of hospital separations)

= (1/β) × *k *× *m*

For a particular match rate α for hospital separations, the corresponding false match rate F for a particular linkage strategy due to chance matches is estimated as

F = False match rate

= C/(Expected number of matches to *m *hospital separations)

= C/(α × *m*)

Prior to matching, neither the numbers of hospital and residential aged care events to be compared nor the number of achieved matches between these events are known. In the absence of such information, the expected numbers of chance matches can be estimated by assuming that hospital separations and entries into residential aged care are independent Poisson processes and then substituting in the relevant expected daily numbers of hospital separations and residential aged care entry events. Furthermore, different strategies for different types of events (for example, different strategies for linking to admissions into residential aged care and for linking to returns to residential care after hospitalisation) can be allowed for by using type-specific daily event rates and match rates for the different types of events.

A maximum for the false match rate F' can be estimated using an anticipated minimum match rate. Therefore, more generally we can estimate the maximum false match rate as

$$F^{\prime }=\frac{\text{Total number of expected chance matches}}{\text{Estimated total number of matches between hospital separations and the different types of aged care entries}}$$

$$\leq \sum_{\text{l}}\left(\lambda_{\text{Hl}}\lambda_{\text{Rl}}/\beta \right)/\sum_{\text{l}}\sum_{\text{t}}\left(\lambda_{\text{Hl}}\alpha_{\text{tl}}\right)$$

$$\leq \sum_{\text{l}}\left(\lambda_{\text{Hl}}\lambda_{\text{Rl}}/\beta \right)/\sum_{\text{l}}\lambda_{\text{Hl}}(\sum_{\text{t}}\alpha_{\text{tl}})$$

where λ_Hl _is the expected daily number of hospital separations of type *l *(for example, of length *l*), *λ *_Rl _is the expected daily number of candidate residential aged care matches for a hospital episode of type *l*, and *α*_tl _is the minimum expected achieved match rate with residential aged care entries of type *t *for hospital episodes of type *l*. The formula could be further generalised by dividing the population into a number of age groups across the age range of interest. The value for F' for a particular linkage strategy can be then derived by substituting in event rates observed in the two separate databases (λ_Hl _calculated from the data on hospital episodes, and λ_Rl _calculated from the data on residential aged care events) and either anticipated match rates or match rates obtained in a feasibility study. Readers requiring detailed technical information on the methodology and statistical theory are referred to Karmel 2004 [38].

From the above, it can be seen that the false match rate depends both on the numbers of events involved in the match comparisons and on the achieved match rate. Consequently, the final achieved false match rate will vary with the strictness of the data matching requirements being used to identify linked events, both in terms of event dates and demographic data. Examples of different event date matching requirements include: insisting that events can only be considered for matching when the hospital separation date and the aged care entry date match exactly; allowing events a day or two apart to be considered as possible matches; and/or requiring matching on both the start and end dates of the hospital event when matching to returns from leave absences for people already in residential aged care. It may also be possible to either broaden or reduce the geographic region used for matching, or to allow differences in date of birth or sex. Allowing variation in the match data when identifying links changes both the expected numbers of events that could be considered for matching (that is, λ_Hl _and λ_Rl_) and the number of achieved matches (α_tl_). Consequently, the above equation can be used to compare a range of matching stratagems that could be applied in different event scenarios or for a range of population sizes.

In practice, when linkage data are recorded consistently on the two data sets being matched, as match criteria are relaxed the match rate increases because of the increase in the number of false matches. However, when there are inconsistencies in the data, relaxing the match strategy (for example, broadening the match region) can lead to identifying previously-missed links. By estimating the number of false matches (as above) in matched data sets derived using different strategies, the trade-off between false matches and matches missed due to inconsistent data can be examined.

## Results

Using the maximum false match rate F' to gauge the utility of the event-based linkage, two aspects of the strategy are examined. First, results are presented for the particular case of linking Australian hospital and residential aged care data. The wider utility of an event-based data linkage methodology, when neither name nor person identifier are available, is then examined (see Discussion). In both cases, the integral role of the level of geography used in the linkage process is unambiguous.

### Linking hospital and residential aged care data

Three types of entry events into residential aged care are considered as candidates for matching to hospital separations: new admissions into residential aged care, episodes of hospital leave (where the resident goes to hospital for a period) and episodes of social leave (where the resident goes to stay with family or friends for a period, during which time they could have an episode in hospital). In 2001–02 in Australia, there were around 900,000 hospital episodes for people aged 65 and over that involved at least one night in hospital and that did not end in either a transfer within the hospital system or death. In the same year there were 91,000 admissions into residential aged care (95% of which were for people aged 65 and over), 70,000 episodes of hospital leave and 54,000 episodes of social leave.

Using daily event rates derived from available hospital morbidity and residential aged care databases (for people aged 65 and over), in conjunction with match rates obtained in the initial feasibility study [13], estimates of the maximum false match rate for a number of event-based linkage strategies were derived. In addition, to gauge the effect of the assumption concerning distribution of birth dates, estimates were derived for two extreme birth date distributions. The results are presented in Table 1.

**Table 1:** Linking hospital and residential aged care events: estimated maximum false match rate for several event-based strategies

	Estimated maximum false match rate (%)	
	
Linkage strategy	Population 10000 (single sex)	Population 35000 (single sex)
Base linkage strategy^(a)^	1.68	5.87
-but allowing up to a two day gap when matching a hospital separation to a new admission into residential aged care (3-date admission matching)	2.67	9.36
-but insisting episodes of hospital leave from residential aged care exactly match on both the start and end of the hospital episode	1.24	4.34
-but excluding matching to episodes of social leave from residential aged care as unlikely	1.05	3.66
-but insisting episodes of hospital leave from residential aged care exactly match on both the start and end of the hospital episode and at the same time excluding matching to episodes of social leave from residential aged care	0.61	2.14
Changing assumption to birth dates spread uniformly over 30 years	0.84	2.93
Changing assumption to birth dates spread across 5-year age groups as per aged care admissions over 30 years	1.10	3.86

(a) Base linkage strategy includes the following assumptions to allow estimation:

• 15 years of birth dates, uniform distribution

• single-date (exact end-date) matching only of separations to residential aged care admissions and to residential aged care hospital leave

• allow separations to match to social leave covering the hospital episode (cover matching).

Calculations use national average hospital separation rates (2000–01) and residential aged care admission and residential aged care leave rates (2001–02) [38]. Estimates take into account the distribution of hospital episodes by length of stay.

Table 1 shows the effects of both relaxing and further constraining a linkage strategy. For example, allowing hospital episodes to match to residential aged care admissions up to two days later increases considerably the number of candidates for matching and so leads to an increase in the estimated maximum false match rate (2.7% compared with 1.7% within a population of 10,000). On the other hand, constraining the strategy by insisting on exact period matching before accepting a match between a hospital episode and a residential aged care hospital leave event results in a reduction of the estimated maximum false match rate (1.2% compared with 1.7% within a population of 10,000). In addition, not allowing links to social leave events also reduces the false match rate as it excludes links that are more likely than others to be erroneous.

For most of the estimates presented in Table 1, birth dates are assumed to be spread across just 15 years. Because this is a much greater concentration of birth dates than actually happens for those aged over 65, this assumption leads to over-estimation of the number of chance matches and therefore the corresponding false match rate. To gauge the importance of the assumption concerning birth dates, the maximum false match rate was also derived assuming that ages are spread uniformly over 30 years results. This results in insufficient concentration of birthdates for the more common age groups, and so leads to an under-estimation of the maximum false match rate. For a population of 10,000, these two extreme birth date distributions give estimated maximum false match rates of 1.68% and 0.84%, respectively (using the base linkage strategy as described in Table 1). If the actual birth date distribution could be used, the maximum false match rate would be between these two estimates: using a more realistic distribution of birth dates results in an estimated false maximum match rate of 1.10% (based on uniform birth date distributions within six 5-year age groups). Therefore, using just a 15-year range for birth dates provides a conservative (high) estimate of the maximum false match rate, so that decisions based on such estimates would lead to cautious recommendations on the use of an event-base matching strategy rather than possibly inappropriate use of the approach.

**Figure 1:**
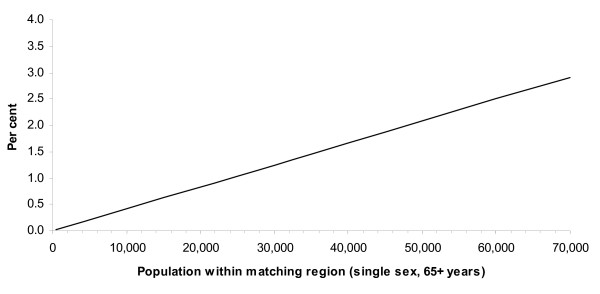
**The effect of population size on the estimated maximum false match rate**. Estimation assumes: • 22 years of birth dates, uniform distribution (compromise distribution). • single-date (exact end-date) matching only of separations to residential aged care admissions. • exact period matching to residential aged care hospital leave. Calculations use national average hospital separation rates (2000–01) and residential aged care admission and residential aged care leave rates (2001–02) [38]. Estimates take into account the distribution of hospital episodes by length of stay.

Noting that the number of events available for matching is simply the product of the event rate and the population size, manipulation of the formula for the false match rate shows that there is a linear relationship between the size of the population within which matching is taking place and the estimated maximum false match rate. For example, in the simplest case where one type of hospital separation is being linked to one type of residential aged care entry, the estimated maximum false match rate can also be written as:

F*' *= r × P/βα

where r is the daily rate (per 1,000 people in the region within which matching is taking place) at which the candidate residential aged care entry event occurs and P is the size of the population in 1,000's (α and β as before). This relationship is evident in both in Figure 1 and Table 1. The utility of the event-based strategy therefore depends on both the event rates and the size of the population within which matching is being considered. If the available geographic information is such that matching can take place within sufficiently small regions so that the numbers of events being compared are small, then the false match rate can be minimised.

When linking Australian hospital morbidity data and residential aged care data the smallest region available for the matching process is postcode area. At the time of the Australian census in 2001, at least 98% of people aged 65 and over lived in postcodes with fewer than 5,000 older women and 5,000 older men. Figure 1 shows that for such small populations, the false match rate would be well under 1% for a range of event-based linkage strategies. If matching requirements are relaxed so that only the first three digits of a postcode are needed to establish a match, census data show that for the resulting larger regions, in 2001, 92% of older women and 98% of older men lived in regions with fewer than 15,000 older people of the same sex; all such regions had fewer than 30,000 people of the same sex. Consequently, using three digit postcode areas and constraining the matching strategy as in Figure 1, the false match rate would be below 1% for a large majority of regions, and under 1.5% for all regions. Such an approach allows matches to be made between records when there are only slight differences in reported postcode. Differences between postcodes for valid matches could be due to either data entry errors in the last digit or to people reporting different but physically close postcodes in the two data collections.

## Discussion

The above results provide information on scenarios for which the event-based linkage strategy could be useful for a particular application–that is, for linking Australian hospital and residential aged care records. However, this theoretical approach can also be used to examine more generally the parameters within which such a linkage strategy could prove useful. In particular, it can allow investigations into the relationships between the false match rate, the rate at which events happen, the achieved (or anticipated) match rate, and the population size within matching is taking place. It can also be used to identify preferred matching strategies when data inconsistencies could lead to missed matches.

For transition systems, movements can be viewed either from the point of view of the source sector (hospital in the current application) or from the point of view of the receiving sector (residential aged care). Consequently, exit events and their match rates in the source sector are related to entry events and their match rates in the receiving sector. As a result, the false match rate can be expressed in two ways. For example, in the simplest case where one type of exit event (any hospital separation in the above analysis) is being linked to one type of entry event (any residential aged care entry), the maximum false match rate can be estimated as either

F*' *= r × P/βα (as given above)

or alternatively as

 F*' *= ρ × P/βa 

where

r is the daily rate (per 1,000 people) at which the candidate entry event occurs

ρ is the daily rate (per 1,000 people) at which the candidate exit event occurs

α is the rate of exit events matching

a is the rate of entry events matching

P is the size of the population (in 1,000's), and

β is the total number of possible birth dates.

**Figure 2:**
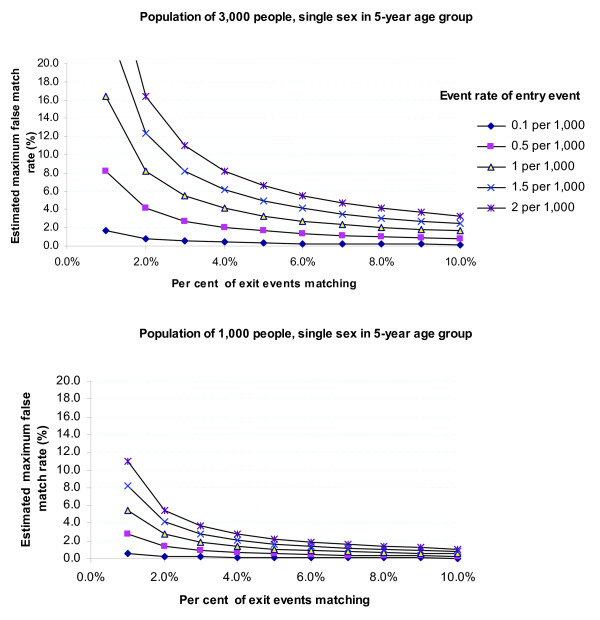
**The estimated maximum false match rate given observed event and match rates: **The estimated maximum false match rate among achieved matches as a function of the match rate among the initial exit events and the occurrence rate of the possibly-related entry events, for two population sizes.

The impact on the false match rate of both event rates and match rates is illustrated in Figure 2 (in terms of the match rate for the exit events and the daily rate of the possibly-related entry events). From both the above equations and Figure 2 a number of points are clear:

• the false match rate declines as the event rate diminishes, that is, as events become rarer;

• the false match rate declines as the number of matches increases; and

• the false match rate increases with population size (as seen before).

In the analysis of movement from hospital into residential aged care presented in Table 1, the daily event rates are 1.2 hospital separations per day per 1,000 people in the population and 0.2 residential aged care entries per day per 1,000 people, with an anticipated match rate of between 5% and 10% for hospital separations which corresponds to a match rate of between 21% and 43% of all residential aged care entry events. These rates place this scenario just above the lowest line (entry event rate of 0.1 per 1,000) on Figure 2. The graphs show clearly that by using some basic information about the transitions, it is possible to measure the likely extent of false matches among achieved matches, and thereby to gauge whether event-based linkage could provide a useful tool for examining movement.

If linkage data are highly accurate, the achieved match rate is affected primarily by the number of false matches, with the number of identified true matches remaining largely unaffected by changes in the linkage strategy. However, in practice true links are either missed or identified depending on the quality of the linkage data. Consequently, the number of achieved matches varies with the precision of the match strategy due to changing numbers of both false and missed matches. For example, a person's four digit postcode may differ in the last digit on the two data sets so that using complete postcode to match would lead to missing the match while using up to the first three digits would allow the match to be identified. On the other hand, using the region defined by the first three digits of postcode would lead to more false matches than using the smaller four digit postcode area. Such differences in match rates are readily observed when developing a linkage strategy (for example, see [13] p15). When two data sets have been linked using a number of strategies that allow different degrees of latitude in the match variables, the estimated false match rate (derived as above) and corresponding true match rate (or positive predictive value) can be used to gauge whether relaxing linkage requirements results in identifying enough previously-missed links to outweigh the additional false links.

**Figure 3:**
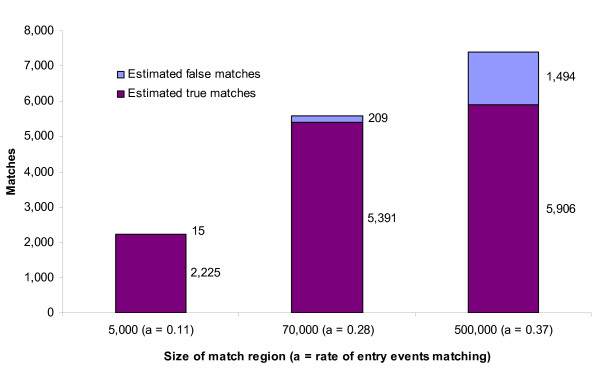
**Example examining the relationship between false links and true links for different linkage strategies**. Figure 3 shows the estimated number of false matches and true matches among achieved matches for links of exit events happening at a rate of 1.2 per day (per 1,000 people) to 20,000 entry events occurring over a year, and demonstrates the change in the entry event match rate as the size of the match region is varied.

Figure 3 illustrates the use of the estimated false match rate for examining the trade-off between false and true links in three linkage strategies. In this example, the match rate of entry events increases from 11% when using relatively small match regions (5,000 people) to 28% when using medium-sized match regions (70,000 people). It then rises up to 37% when using large match regions (500,000 people). These figures are similar to those observed in the initial feasibility study for the event-based matching method when matching hospital separations for women aged 65 and over to residential aged care admissions within the person's postcode of usual residence, Statistical Local Area of usual residence and State of usual residence, respectively [13]. In this example, using the small match region results in very few false links–estimated at just 0.7%. However, comparison with the matches obtained based on the larger regions shows that, while very accurate, using the small regions results in missing a large number of matches. By increasing the match region to 70,000 it is estimated that among the new matches true matches outnumber false matches by 16 to one, with the number of true matches more than doubling while the false match rate remains below 5%. On the other hand, changing from medium to large match regions (500,000) adds more than twice as many false matches as true matches (an estimated 1,285 versus 515) resulting in a false match rate of 20% among the achieved matches.

In this example, using the theoretical analysis to estimate the number of false matches, the researcher could see that the increase in matches observed when going from the small to medium-sized match regions was driven by the identification of previously-missed matches, while the substantial increase in matches observed when going from the medium to large match regions was largely the result of false matches. Depending on the accuracy requirements of the study, the researcher could then decide either to use the linkage strategy based on the medium-sized match region, or to test other region sizes for matching. This example illustrates the value in knowing whether increases in match rates are likely to be the result of false matches or the identification of missed matches.

## Conclusion

From a policy perspective, knowledge of the relationship between hospital and residential aged care is vital for the optimal care of older people. While, in Australia, considerable data are available on both sectors separately, the problem is to determine whether the two corresponding data sets can be reliably linked–thereby generating a linked database which could be used for analysis of patterns of movements–even though neither name nor a common person identifier are available on both data sets. Analysis of the event-based linkage strategy presented in this paper demonstrates that such linkage is possible: estimates indicate that the linkage strategy results in an acceptably low prevalence of false matches, and so can be used to derive a data set useful for investigating the hospital-residential aged care interface.

A key factor determining whether or not the strategy could be used is the number of candidate matches being considered for an exit event: if this is too high the probability of chance matches becomes too great and the false match rate increases accordingly. A further consideration is the likelihood of missing links due to variations in reporting data items. For event-based strategies the number of candidate matches is driven by both the rate at which events occur and the size of the population within which matches are to be made. By appropriate choice of the population groups used for linkage and/or the strictness of the matching stratagem, the number of chance matches can be kept to an acceptably low level without increasing unduly the number of missed links so that the linked data set could be useful for many types of analyses.

There is an increasing policy emphasis on looking at service provision from a whole-of-person viewpoint, with a longitudinal perspective being preferred. Therefore, the ability to examine transitions between services becomes key for policy-relevant research. As a consequence, capacity to link program-specific data sets takes on added significance. Analysis in this paper shows that in a wide range of situations linkage of transition events across data sets is possible even if name or person identifiers are not available.

Whether the linked data set derived using the event-based strategy is sufficiently complete to allow examination of flows as well as patterns of movements depends largely on the quality and compatibility of the linkage variables. This paper has outlined a theoretical approach that allows the estimation of the false match rate in a proposed linkage strategy and as a consequence examination, to some extent, of the trade-off between false and missed matches–an issue faced by many linkage strategies. The methods in this paper provide researchers with tools that can help them firstly to decide whether event-based linkage could be useful, and secondly to identify preferred match criteria once matching between two specific data sets is being undertaken. The overall relationship between false and missed links in the context of movements of linking hospital to residential aged care data will be explored in a subsequent study that compares the event-based linkage strategy with a full name-based linkage.

## Abbreviations

AIHW: Australian Institute of Health and Welfare; COAWG: Care of Older Australians Working Group; HCOASC: Health Care of Older Australians Standing Committee.

## Competing interests

The author(s) declare that they have no competing interests.

## Authors' contributions

RK investigated and developed the event-based matching method. DG designed and developed the event-based matching method to link related events for individuals without requiring name or a person identifier, and gave final approval for the article as published.

## Pre-publication history

The pre-publication history for this paper can be accessed here:

http://www.biomedcentral.com/1472-6963/7/154/prepub
